# Short-term intrinsic connectivity changes induced by cognitive exertion in healthy participants

**DOI:** 10.3389/fnins.2026.1781534

**Published:** 2026-04-20

**Authors:** Leighton Barnden, James Baraniuk, Maira Inderyas, Sonya Marshall-Gradisnik, Kiran Thapaliya

**Affiliations:** 1 National Centre for Neuroimmunology and Emerging Diseases, Griffith University, Gold Coast, QLD, Australia; 2 Department of Medicine, Georgetown University, Washington, DC, United States

**Keywords:** cognition, fatigue, fMRI, functional connectivity, independent component analysis, internetworks, Stroop task

## Abstract

**Introduction:**

Changes in brain intrinsic connectivity on the timescale of minutes, as provoked by a cognitive task, have not been well documented.

**Methods:**

A total of two 7.5-min 7 Tesla functional MRI (fMRI) scans (Run 1 and Run 2), separated by 90 s, were acquired for 23 healthy participants during cognitive exertion induced by the Stroop color–word interference task. Independent component analysis (ICA) of the paired Run 1 and Run 2 fMRI acquisitions identified components with distinct spatial and temporal signatures.

**Results:**

The spatial extent of the ICA components coincided with hubs of the brain’s intrinsic networks. In addition, these components correlated with brain regions from other networks, thereby defining inter-network connectivity. Run 1 and Run 2 showed significantly different patterns of connections (p-FWE < 0.01) across 10 ICA-identified intrinsic networks and 20 inter-networks. Connectivity in Run 2 was higher in 12 nodes and lower in eight nodes, indicating dynamic changes during the task response. Overall, the right angular gyrus/supramarginal gyrus and the right frontal pole regions of the ventral attention network showed greater activity in Run 1, but activity shifted to the fusiform gyrus, supplementary motor area (SMA), and precentral and postcentral gyrus nodes in Run 2. Response times (RTs) and Stroop test accuracy did not change between runs in these healthy participants.

**Conclusion:**

Inter-network connectivity indicated that surveillance and task oversight nodes were required early in learning how to complete the Stroop task (Run 1), but these were replaced by object recognition and more automatic responses in Run 2. These findings define inter-networks that are sensitive to cognitive exertion and provide a framework for understanding cognitive dysfunction.

## 1 Introduction

Most functional MRI (fMRI) studies are designed to yield a temporal ‘snapshot’ or average of functional connectivity. Studies of short-term adaptation in connectivity patterns during cognitive exertion are limited. We evaluated differences between two consecutive 7.5-min fMRI scans to assess the evolution of connectivity during task completion. The 15-min duration is unique and innovative because it allows inferences about changes in cognitive processing over a longer time period than previously examined, providing a foundation in healthy participants for comparisons with chronic fatigue and other disease states. Control participants were used to establish a foundation for future comparisons with disease groups (Yeon et al., 2024).

The resting brain shows “intrinsic” networks of correlated brain region activities that contribute to cognitive function (Yeo et al., 2011). The salience (SA) network, central executive (CE, frontoparietal) network, and default mode network (DMN) are regarded as ‘core’ intrinsic networks (Menon, 2011). The salience network controls whether the DMN or CE network is dominant based on the level of internal versus extra-personal stimuli (Menon and Uddin, 2010). Other intrinsic networks include the sensorimotor, language, visual, dorsal attention, and cerebellar networks. Each of these *intra*-networks can form novel correlations with nodes from other intrinsic networks, which are termed *inter*-network connections. Inter-network connectivity has been shown to be age-dependent (Aboud et al., 2018) and abnormal in schizophrenia (Rong et al., 2023), late-life depression (Li et al., 2017), and in individuals with brain tumors (Metwali et al., 2020). Previous studies have utilized structural MRI of network hubs (Aboud et al., 2018) and resting-state functional connectivity (Rong et al., 2023), with further insights provided by independent component analysis (ICA; Li et al., 2017).

Here, we test for differences in blood-oxygenation-level–dependent (BOLD) responses between consecutive 7.5-min fMRI scans acquired during the Stroop color–word interference task (Stroop, 1935). The fMRI scans were acquired on a 7 Tesla research MRI scanner. The general class of neuropsychological interference effects provokes prolongation of response times (RTs) when irrelevant features of a task “interfere” with task-relevant elements (Peterson et al., 2002). The two fMRI runs were separated by 90 s. We hypothesized that the first run would reveal processes related to learning and solving the new task, while Run 2 would indicate evolving connections related to fatigue, conflict adaptation, or practice effects. In our case, two words in different ink colors were presented, where the words and colors were congruent or incongruent. The natural process of reading the incongruent word interfered with the task of color matching.

Network processes invoked by the Stroop task include selective attention for task execution, mediated by the dorsolateral prefrontal cortex (DLPFC) of the frontoparietal executive control network (Kalanthroff and Henik, 2014; Perrotta et al., 2021). The lateral prefrontal cortex is proposed to deconvolve the color–word interference in the Stroop task (Banich, 2019; Bush et al., 1998), while the posterior DLPFC establishes the appropriate rules to complete the task (Banich et al., 2000). The left and right DLPFC monitor task conflict and adaptation (Milham et al., 2003). The anterior cingulate cortex (ACC) contributes to error checking by monitoring bilateral DLPFC function (Banich et al., 2000; Carter et al., 2000; Pardo et al., 1990; Gruber et al., 2002). The posterior dorsal ACC activates the medial supplementary motor area (SMA), leading to the ultimate button press that marks the end of each test (Milham et al., 2003). Processing of the challenging incongruent stimulus activates the left intraparietal lobe and bilateral extrastriate visual cortex (Bianco et al., 2021). Distracting stimuli may stimulate the right ventral attention network, activating the right temporal parietal junction, inferior frontal gyrus, and insula (Ghahremani et al., 2015; Aron et al., 2014). The right anterior insula is closely connected to the dorsal ACC (dACC) within the salience network that may recognize the interference of the incongruent condition and recruit inhibitory control mechanisms through the dACC, SMA, and separate connections to the right putamen to facilitate planning for flexible, goal-oriented, adaptive behavior (Bari and Robbins, 2013; Deng et al., 2018).

We predicted that Run 1 (0 to 7.5 min) would engage these networks, while Run 2 (9.0 to 16.5 min) would show altered inter-network connectivity when analyzed using the highly sensitive and specific paired independent component analysis (ICA). We hypothesized that significant differences in inter-network connectivity during the Stroop task from baseline would establish a basis for understanding fatigue, adaptive or practice effects, and mechanisms of cognitive dysfunction. This was reasonable because other task-repetition studies on a similar timescale have reported learning-related changes in functional connectivity within the frontoparietal and dorsal and ventral attention networks (Yeon et al., 2024; Park et al., 2024; Kardan et al., 2023; Hofstetter et al., 2017).

## 2 Methods

### 2.1 Participants

The study was approved by the Griffith University Human Research Ethics Committee (2022/666), and all methods were performed in accordance with their guidelines and regulations and the Declaration of Helsinki. Written informed consent was obtained from all individuals. This cross-sectional investigation was conducted at the National Centre for Neuroimmunology and Emerging Diseases (NCNED) on the Gold Coast, Queensland, Australia. Eligible participants were referred by medical practitioners and assessed using the NCNED research questionnaire for fatigue-affected individuals, which recorded the severity of post-exertional malaise, cognitive disturbances, immune manifestations, thermoregulatory complaints, gastrointestinal symptoms, urinary frequency, body pain, and sleep disturbances. Medical history was requested to identify comorbid manifestations or exclusionary diagnoses, including mental illness, malignancies, autoimmune disorders, neurological disorders, or cardiovascular diseases. Female participants were neither pregnant nor breastfeeding. Recruitment took place between 2022 and 2024.

### 2.2 The Stroop task

The Stroop color–word task (Stroop, 1935) was adapted to investigate attention and concentration performance and has been shown to effectively demonstrate dysfunction in myalgic encephalomyelitis/chronic fatigue syndrome (ME/CFS) and long COVID (Baraniuk et al., 2024). Each Stroop trial displayed two colored words. The upper word was RED, BLUE, YELLOW, GREEN, or XXXX and was presented in red, blue, yellow, or green on a black background. The lower word was RED, BLUE, YELLOW, or GREEN, printed in white on a black background. Participants were asked to decide whether the *color* of the upper word matched the *meaning* of the lower word and press one of two buttons on a handpiece to respond ‘yes’ or ‘no’. A confounding factor was introduced when the upper word did not match its color.

The Stroop task fMRI was divided into four conditions, three of which were trials: *Neutral,* in which the upper word was XXXX; *congruent* (Congr), when the upper word’s color matched the lower word’s meaning; and *incongruent* (Incon), when the color of the upper word differed from the lower word’s meaning. The incongruent condition is considered more challenging because an inhibitory element is required to overcome the natural impulse to read the upper word. The automatic tendency to read the upper word, rather than inspecting its color, creates interference in the color identification task. The fourth condition, *rest*, was the period between a trial response and the onset of the next trial. During this ‘rest’ condition, a fixed, stationary cross appeared on the screen for a duration randomized between 3 and 12 s.

In each task fMRI, a total of 60 Stroop trials were randomly distributed across each 7.5-min acquisition, with an average interstimulus interval of 7.5 s. Of these trials, 40% were incongruent, 30% were congruent, and 30% were neutral. For each trial type, stimulus onset and response times were recorded, and the difference was calculated as the response time. Accuracy for each run was calculated as the number of correct responses divided by the total number of responses.

Each participant received a brief Stroop training session immediately before the commencement of the 7 T fMRI scans. Participants were shown color printouts of Stroop test screens, including examples of congruent, incongruent, and neutral trials, and the correct responses were explained. Hands-on practice with the task was conducted in the scanner.

The dependence of response time on the preceding stimulus was investigated by searching the Stroop records for the prestimulus–stimulus pairs of interest and retaining the corresponding stimulus response times.

Data were not separated by congruent, incongruent, and neutral stimulus responses for this analysis.

### 2.3 MRI acquisition

The study acquired two 7.5-min fMRI scans, separated by 90 s. Both scans were acquired sagittally for each participant during the cognitive Stroop color–word interference task.

fMRI image volumes were acquired on a 7 T whole-body MRI research scanner (Siemens Healthcare, Erlangen, Germany) using a 32-channel head coil (Nova Medical Wilmington, USA). For each fMRI session, 225 volumes were acquired using a multiband echo-planar imaging (EPI) pulse sequence developed at the University of Minnesota (Auerbach et al., 2013), with 80 sagittal slices, multiband factor = 3, TR = 2000 ms, TE = 22.4 ms, flip angle = 70°, acquisition matrix = 192 × 192, and voxel size = 1.25 mm^3^. During the acquisition of these 225 fMRI volumes, the participant responded to a sequence of Stroop color–word tests (Leung et al., 2000).

An anatomical image was acquired using Siemens T2 ‘SPACE’ optimized 3D fast spin-echo (T2wSE) sequences, with TR = 3,200 ms, TE = 563 ms, and variable flip angle scans on an adjacent 3 T scanner on the same day. The acquisition time was 5:44 (min:sec). These scans employed an optimized variable flip angle sequence (Siemens SPACE) to yield a ‘true 3D’ acquisition in a shortened time. Their ‘contrast equivalent’ TE is comparable to that of standard T2wSE sequences (Busse et al., 2006), although the signal is also influenced by T1 relaxation (Mugler, 2014), possibly more so than for T2wSE. These T2 ‘SPACE’ images were sagittal with a pixel size of 0.88 × 0.88 × 0.9 mm. T2 ‘SPACE’ was chosen for the anatomical scan because the spin-echo sequence renders it resistant to the magnetic field-induced distortions that can affect conventional gradient-echo MPRAGE sequences (Bushberg et al., 2002), particularly in the brainstem.

### 2.4 MRI processing

To maximize sensitivity to differences between Run 1 and Run 2, we performed paired ICA on their two BOLD time series. By defining two BOLD time series, one for each run, with corresponding subjects, CONN defaulted to a paired analysis. ICA aggregated the full spatial and temporal datasets of both groups and identified independent components, each with a unique temporal and spatial signature for each participant. We chose 15 components as a compromise between the number of potential intrinsic networks and the complexity of their nodes and connections. A single independent component may include multiple spatially separated locations, which, because they have a common correlated temporal signature, can be regarded as functionally connected. ICA components were assigned to reference (resting-state) intrinsic networks based on correlations with their temporal signatures. Connectivity maps for each participant were generated for each component’s spatial extent, and statistical inference of paired cohort differences in inter-network connectivity between the two runs was performed and expressed as clusters of voxels.

All MRI data were analyzed using CONN (Whitfield-Gabrieli and Nieto-Castanon, 2012; RRID: SCR_009550), release 20.b (Nieto-Castanon, 2020), and the SPM (Penny et al., 2011; RRID: SCR_007037), release 12.7771, toolbox.

Preprocessing: Functional and anatomical data were preprocessed using CONN’s flexible preprocessing pipeline (Nieto-Castanon, 2020), which included realignment with correction for susceptibility–distortion interactions, slice-timing correction (STC), outlier detection, direct segmentation, and MNI-space normalization, smoothing, and removal of initial scans. Functional data were realigned using the SPM realign and unwarp procedure (Andersson et al., 2001), where all scans were coregistered to a reference image (the first scan of the first session) using a least squares approach and a six-parameter (rigid body) transformation (Friston, 1995) and resampled using B-spline interpolation to correct for motion and magnetic susceptibility interactions. Temporal misalignment between different slices of the functional data (acquired in interleaved Siemens order) was corrected using the SPM slice-timing correction (STC) procedure (Sladky et al., 2011), with sinc temporal interpolation to resample each slice’s BOLD time series to a common mid-acquisition time. Potential outlier scans were identified using ART, defined as acquisitions with framewise displacement above 0.9 mm or global BOLD signal changes exceeding 5 standard deviations (Power et al., 2014). A reference BOLD image was computed for each participant by averaging all scans, excluding outliers. Functional and anatomical data were normalized into standard MNI space, segmented into grey matter, white matter, and CSF tissue classes, and resampled to 1.25 mm isotropic voxels using a direct normalization procedure (Calhoun et al., 2017) with the SPM unified segmentation and normalization algorithm (Ashburner and Friston, 2005; Ashburner, 2007) and the default IXI-549 tissue probability map template. Functional data were smoothed using spatial convolution with a Gaussian kernel of 4 mm full width at half maximum (FWHM). Finally, the first five volumes in each functional run were removed.

Denoising: Functional time series were then denoised using CONN’s standard denoising pipeline (Nieto-Castanon, 2020), which included regression of potential confounding effects characterized by motion parameters and their first-order derivatives (12 factors; Friston et al., 1996), outlier scans (Power et al., 2014), white matter time series (5 CompCor noise components), CSF time series (5 CompCor noise components), and linear trends (2 factors) within each functional run, followed by high-pass frequency filtering of the BOLD time series above 0.008 Hz. CompCor (Chai et al., 2012) noise components within white matter and CSF were estimated by computing the average BOLD signal and the largest principal components orthogonal to the BOLD average, motion parameters, and outlier scans within each participant’s eroded segmentation masks. Based on the number of noise terms included in this denoising strategy, the effective degrees of freedom of the BOLD signal after denoising were estimated to range from 50.5 to 58.4 (average 57.7) across all participants (Nieto-Castanon, 2025).

Based on the amplitude of their deformation during spatial normalization, four of the 27 original participants failed QA and were excluded from further analysis.

First-level analysis: The BOLD signal from every time point and voxel in the brain was concatenated across all participants and both Run 1 and Run 2 along the temporal dimension. Group-level independent component analysis (group-ICA; Calhoun et al., 2001) was performed on this fMRI data entity to estimate 15 independent components, each with a distinct temporal signature and spatial map. A singular value decomposition of the z-score-normalized BOLD signal (participant-level SVD), with 64 components separately for each participant, was used as a participant-specific dimensionality reduction step. The dimensionality of the concatenated data was further reduced using a singular value decomposition (group-level SVD) limited to 15 components, and a fast-ICA fixed-point algorithm (Calhoun et al., 2001) with a hyperbolic tangent (G1) contrast function was used to identify spatially independent group-level networks from the resulting components. Finally, GICA3 back-projection (Erhardt et al., 2011) was used to compute 3D connectivity maps associated with each component for each individual participant.

Group-level analyses were performed using a general linear model (GLM; Nieto-Castanon, 2020). For each individual voxel, a separate GLM was estimated, with the first-level connectivity measure (correlation coefficient) at that voxel as the dependent variable and paired Run 1 and Run 2 groups—defined via participant-level acquisition identifiers—as independent variables. A covariate was included to effectively exclude four of the 27 participants who failed spatial normalization QA. Voxel-level connectivity hypotheses were evaluated using multivariate parametric statistics with random effects across participants and sample covariance estimation across pairs of measurements. Inferences were performed at the level of individual clusters (groups of contiguous voxels). Cluster-level inferences were based on parametric statistics derived from Gaussian random field theory (Worsley et al., 1996; Nieto-Castanon, 2020). The results were thresholded using a combination of a cluster-forming voxel-level threshold of *p* < 0.001 and a false discovery rate (FDR)-corrected cluster-size threshold of p-FDR < 0.05 (Chumbley et al., 2010). This yielded spatial distributions as T-statistic maps highlighting brain areas with different connectivity between Run 1 and Run 2 to the independent component’s spatial map.

### 2.5 ICA components and intrinsic networks

**Figure 1:**
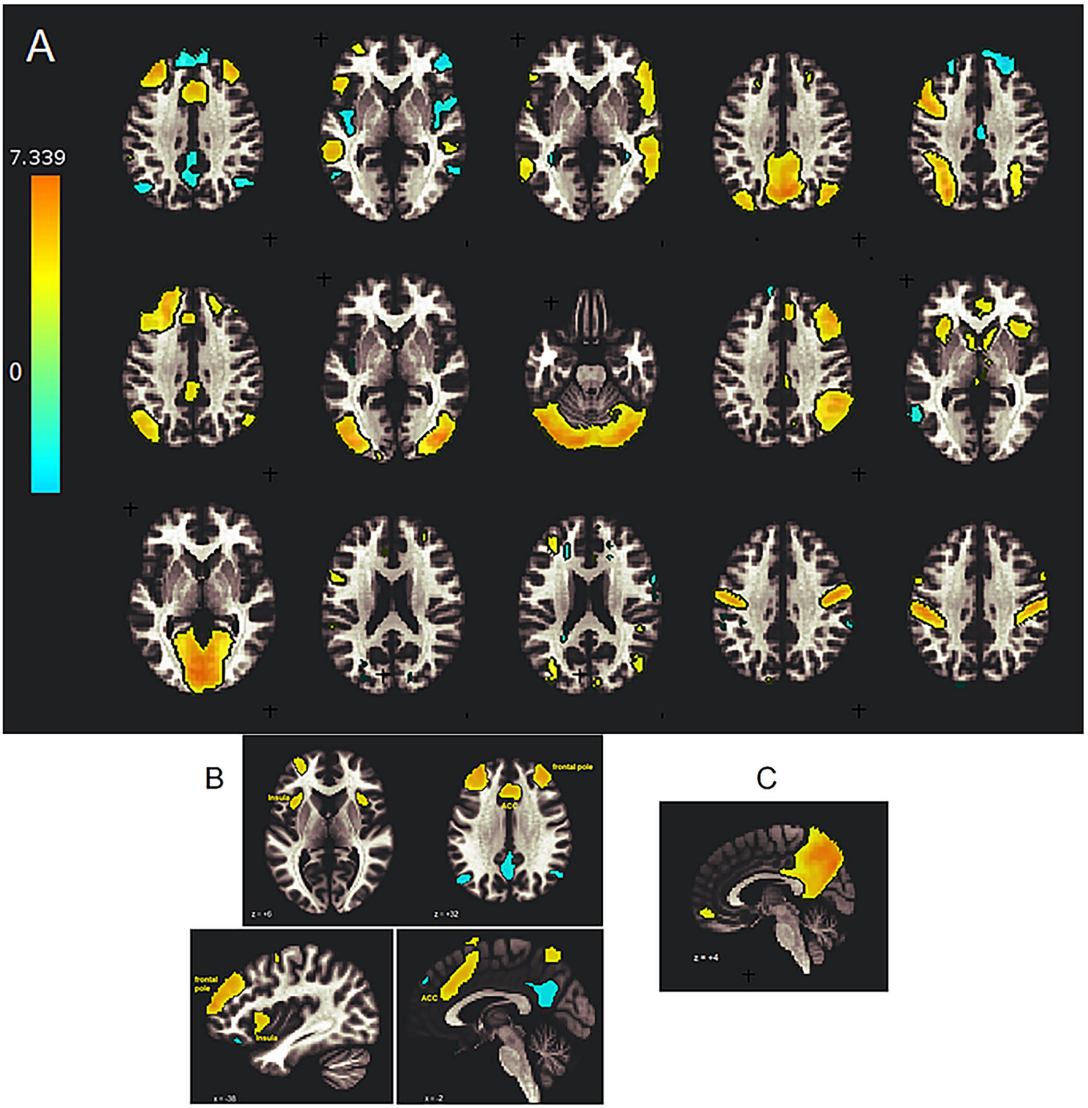
Spatial maps of 15 ICA components from group-ICA analysis of aggregated Run 1 and Run 2 BOLD fMRI data from 23 healthy participants, shown on selected transaxial MRI slices. A total of four participants failed QA and were excluded. Colored voxels indicate the statistical inference (*Z*) of the group ICA relative to the GLM analysis of individual participant ICA components. Red–yellow voxels indicate significantly greater group connectivity (*Z* > 2.5), and blue voxels indicate significantly lower group connectivity (*Z* < −2.5) relative to individual participants. **(A)** The top row shows ICA components #1–#5, the second row shows #6–#10, and the third row shows #11–#15. The best-fit intrinsic networks were assigned as follows: ICA#1: Salience, #2: Language, #3: Language, #4: posterior DMN, #5: CE, #6: CE + DMN, #7: Visual, #8: Cerebellar, #9: CE, #10: Salience, #11: Visual, #12: Salience+DMN, #13: DMN, #14: Sensorimotor, and #15: Dorsal Attention+ sensorimotor. Transaxial sections are shown for MNI *z*-coordinates = 36, 0, 0, 36, 36; 36, 0, −24, 36, 0; and 0, 20, 20, 36, 36, respectively. **(B)** Multiple sections of ICA component #1 demonstrate the 3D extent of the salience network hubs in the bilateral insula, dorsal ACC, and dorsolateral prefrontal cortex. The upper sections are shown at MNI *z* = +6 and +32, and the lower pair at *x* = −36 and +2 mm. **(C)** Peri-midline section of ICA component #4 showing DMN hubs in the posterior cingulate cortex (PCC), precuneus, and medial prefrontal cortex.

Multiple spatially separated areas (clusters) in a single ICA component share the same temporal signature and can be said to exhibit connectivity. CONN identified the ICA components with intrinsic networks according to their correlation with template temporal signatures. Each component was classified as belonging to one of eight intrinsic networks: Default mode network (DMN), salience, frontoparietal (central executive), sensorimotor, language, visual, dorsal Attention, or cerebellar, according to its correlation with resting-state template temporal signatures. Some ICA components involved two networks, and some networks involved multiple ICA components. We show Z-maps for ICA components associated with these intrinsic networks in Figure 1. One representative section is shown for each component in the upper panel, while additional sections in Figures 1B,C illustrate the 3D extent of the salience network and DMN hubs.

Here, we visualized clusters with different connectivity between Run 1 and Run 2 to an ICA component on surface views of the brain using semi-inflated white matter surfaces. These views showed the intersection of a cluster with the surface, which sometimes caused a single cluster to appear as separated areas on the surface.

## 3 Results

The 27 healthy controls (19 female participants) had a mean age of 37.9 years (SD 10.9). The mean BMI was 24.3 ± 2.6 (mean ± SD).

### 3.1 Stroop task response times and accuracy

Table 1 shows the mean RTs for each of the three Stroop task stimuli in both Run 1 and Run 2. Differences between the runs were not significant.

**Table 1:** Stroop task results.

	Metric	Response time (seconds)			Stroop
		Incongruent	Congruent	Neutral	Effect
Run 1	Mean	1.574	1.314	1.328	0.171
Run 2	Mean	1.415	1.316	1.248	0.073
Run 1 vs. Run 2	*p*	ns	ns	ns	ns

Mean RTs are listed for incongruent, congruent, and neutral Stroop stimuli during Run 1 and Run 2. Stroop effects were calculated. Statistical inference (*p*) was not significant for between-run comparisons (two-sided; ns indicates *p* < 0.05).

### 3.2 Independent component analysis

**Figure 2:**
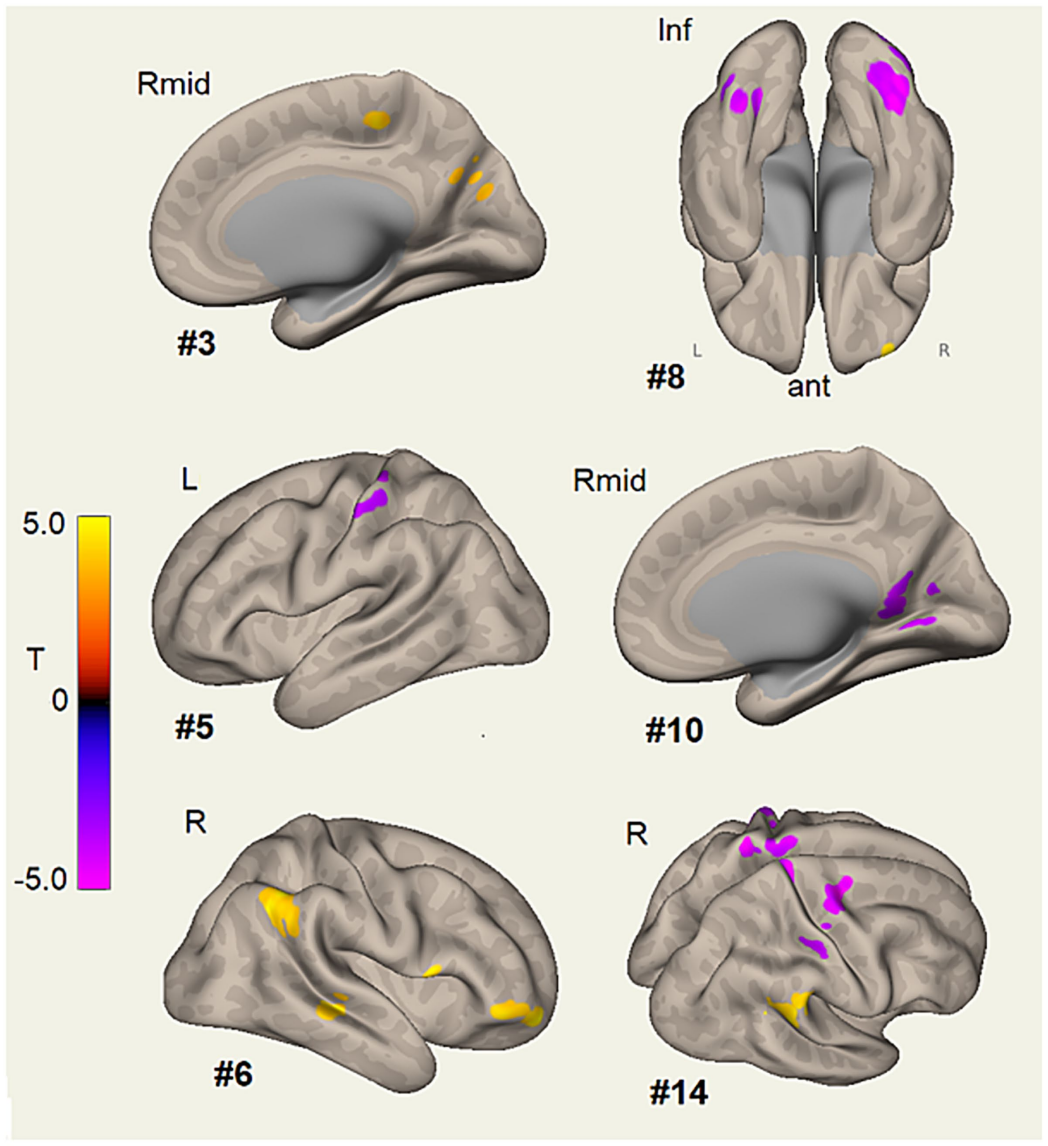
Clusters of voxels showing different connectivity between Run 1 and Run 2 corresponded to ICA components #3, #5, #6, #8, #10, and #14. Yellow–orange voxels indicate *T* statistics for Run 1 > Run 2 connectivity, while pink–purple voxels indicate *T* statistics for Run 1 < Run 2 connectivity. Table 2 lists their statistical inference. The clusters of #3 connect to the language network, #5 to the central executive (CE) network, #6 to the composite CE and DMN, #8 to the cerebellar network, #10 to the salience network, and #14 to the sensorimotor network (see Figure 1). The color bar represents voxel *T* values and indicates the polarity of the difference. Refer to Table 2 for details. Clusters are shown where they intersect the semi-inflated white matter surface. For ICA #8, connectivity differences were observed in the occipital fusiform gyrus. Components #6 and #14 showed elevated Run 1 connectivity for the right angular gyrus (#6 more superior). Surface views are as follows: Left midline for #3 and #10, left for #5, right for #6, inferior for #8, and right–superior for #14, illustrating the bilateral extent of the mid-postcentral and precentral cluster.

Figure 1A shows spatial maps of the 15 components from the ICA analysis of the aggregated 225 BOLD volumes from Run 1 and Run 2 across 23 participants (four of the original 27 participants failed spatial normalization QA and were excluded). Each component exhibited a distinct temporal and spatial (shown) signature. Figure 1B shows the extent of salience network connectivity, with elevated connectivity shown in yellow and reduced connectivity shown in blue, based on GLM analysis of the single-subject components. Figure 2 shows elevated salience network (ICA #1) connectivity in the anterior cingulate cortex (ACC), bilateral anterior insula, and frontal pole regions, along with reduced connectivity in posterior DMN nodes. This also highlights the limitation of representing network hubs with a single section in Figure 1A. Choosing 15 ICA components allowed the separation of left (ICA 5 and 6) and right (ICA 9) nodes of the CE network, two language networks (ICA 2 and 3), and two networks in the occipital visual system (ICA 7 and 11). These networks are consistent with previously reported activation patterns for the Stroop task (Peterson et al., 2002; Kalanthroff and Henik, 2014; Perrotta et al., 2021; Banich, 2019; Bush et al., 1998; Banich et al., 2000; Milham et al., 2003; Carter et al., 2000; Pardo et al., 1990; Gruber et al., 2002; Bianco et al., 2021; Ghahremani et al., 2015; Aron et al., 2014; Bari and Robbins, 2013), with the addition of a cerebellar network (ICA 8).

### 3.3 Inter-networks

Table 2 characterizes each component according to the intrinsic network assigned by CONN. The ‘r’ column lists the highest temporal correlation with CONN’s template networks. Voxel clusters showing differences in connectivity to an ICA component between Run 1 and Run 2 define the inter-network effects. Connectivity in Run 2 was classified as either higher or lower relative to Run 1 based on the Family Wide Error (p-FWE). Cluster size in voxels (k) and center-of-mass coordinates in the MNI system were reported.

**Table 2:** Inter-networks.

ICA #	Network	r	Cluster	High run	Center MNI	k	p-FWE
1	Salience	0.44	-				
2	Language	0.25	-				
3	Language	0.31	Precentral mid	1	+4,-30,+52	91	0.006
4	DMN	0.56	Parietal sup R	2	+30,-50,+70	53	0.037
5	CE	0.18	Postcentral L	2	−52,-24,+52	96	0.001
6	CE &	0.16	Angular R	1	+54,-50,+44	276	<10^−6^
	DMN	0.15	Fron pole R	1	+46,+44,-12	81	0.0008
			Fron pole R	1	+34,+60,-10	65	0.004
			Temp middle post R	1	+52,-28,-8	57	0.009
7	Visual	0.53	Occipital Lat sup R	1	+30,-60,+50	90	0.0003
8	Cerebellar	0.44	Occipital fusiform R	2	+36,-70,-14	179	<10^−6^
			Fron pole R	1	+34,+58,-2	94	0.0003
			Occipital fusiform L	2	−32,-68,-12	82	0.0008
9	CE	0.34	Frontal pole R	2	+28,+42,+42	78	0.005
			Occipital Lat sup R	2	+36,-76,+34	57	0.03
10	Salience	0.068	Precuneus	2	+4,-56,+10	141	0.000007
11	Visual	0.56	-				
12	Salience & DMN	0.054 0.048	-				
13	DMN	0.091	-				
14	Sensorimotor	0.53	SMG post / Angular R	1	+58,-40,+30	190	<10^−6^
			Postcentral+precentral mid	2	+6,-36,+62	165	<10^−6^
			Precentral R	2	+30,-12,+66	125	0.00003
			Postcentral L	2	−44,-24,+58	58	0.01
15	Dorsal Attention	0.45	Fron sup L	2	−22,+20,+56	54	0.04
	+ Sensorimotor	0.24	Postcentral R	2	+60,-10,+34	51	0.05

The 15 ICA-defined intrinsic networks (listed in the ‘Network’ column) included 20 clusters showing significantly different connectivity between Run 1 and Run 2 (p-FWE < 0.01). The ‘r’ column indicates the network temporal correlation with CONN’s template intrinsic network. Some networks (e.g., #10 and #12) were weakly correlated (*r* < 0 0.10). The ‘High Run’ column lists the run with greater connectivity, and ‘k’ represents cluster size in voxels. A total of five networks (#1, 2, 11, 12, and 13) showed no differences in connectivity between Run 1 and Run 2 at other brain sites. However, 20 cluster connections differed between runs. A total of two right angular cortex–SMG inter-network clusters (see #6, #14) exhibited very high statistical inference (p-FWE < 10^−6^) for a Run 2 connectivity deficit. Clusters were defined using a voxel-wise threshold of *p* < 0.001. CE, central executive (also known as frontoparietal); DMN, default mode network; FWE, family wise error; SMG, supra marginal gyrus; Lat, lateral; L, left; R, right.

ICA components 1, 2, 11, 12, and 13 showed no inter-network correlation changes, suggesting steady function throughout the Stroop task. The remaining networks were associated with 20 nodes that showed significant differences between Run 1 and Run 2. Run 1 showed greater connectivity for eight nodes, while Run 2 showed greater connectivity for 12 nodes.

Run 1 was higher connectivity for ICA 6 (CE and DMN), with significantly greater connectivity to the right angular gyrus/supramarginal gyrus and the right frontal pole (p-FWE < 1×10^−6^) compared to Run 2. Run 1 also exhibited higher connectivity for a precentral gyrus node with ICA 3 (language) and the right lateral superior occipital region with ICA 7 (visual).

Conversely, nodes in Run 2 showed higher connectivity for ICA 4 (DMN), ICA 5 (CE), ICA 9 (CE), 1 ICA 0 (Sal), and ICA 15 (DAN and sensorimotor).

Cerebellar (ICA 8) and sensorimotor (ICA 14) networks showed a mixture of changes, suggesting active switching of correlations during the course of the Stroop task. ICA 14 (sensorimotor) connectivity was higher in Run 1 for the right SMG/angular gyrus but higher in Run 2 for the midline and bilateral precentral and postcentral gyri (p-FWE < 1×10^−6^ for midline). This shift suggests that the analytical integrative functions of the right SMG were switched to sensorimotor activities of the supplementary motor area (SMA) and the upper limb during the evolution of the Stroop task response. ICA 8 showed higher connectivity with the right frontal pole in Run 1, which shifted to higher connectivity with the right occipital fusiform gyrus region in Run 2.

Overall, Run 1 was associated with higher connectivity for the SMG/angular gyrus (ICA 6 and 14) and the right frontal pole (ICA 6 and 8). These nodes belong to the ventral attentional network (Bernard et al., 2020). In contrast, Run 2 showed greater connectivity for precentral and postcentral nodes (ICA 5, 14, and 15) and the fusiform gyrus region in cerebellar ICA 8. These changes suggest a progression from task oversight by the ventral attention network to color–word object recognition through interactions with the cerebellar fusiform gyrus (Palejwala et al., 2020), followed by task preparation (Deecke and Kornhuber, 1978) and completion via the SMA (Cunnington et al., 2003) and sensorimotor cortex.

## 4 Discussion

These results are derived from a unique dataset of paired 7.5-min fMRI time series, acquired 90 s apart during the cognitive effort of the Stroop color–word task. The repeat fMRI (Run 2) reflects the consequences of the cognitive effort performed in the baseline scan (Run 1). Differences may reflect fatigue (Run 2 < Run 1) and/or training effects (Run 2 > Run 1). It is remarkable that paired Independent Component Analysis (ICA) of BOLD time series in this healthy cohort was validated by the colocalization of ICA component spatial maps with hubs of the brain’s intrinsic networks.

ICA is an advanced, comprehensive analysis technique that operates on the aggregated BOLD data from all cohort participants across both runs. It provides a simplified representation of the complex connectivity within the human brain. Each ICA component has a distinct spatial and temporal signature. We correlated the temporal signature of each ICA component with the BOLD time series of all voxels in the rest of the brain for each participant and examined cohort differences between Run 1 and Run 2. Significant voxel clusters defined inter-network connections, with some showing greater connectivity in Run 2 than in Run 1, and others showing reduced connectivity in Run 2.

The changes in inter-network connectivity between Run 1 and Run 2 suggested an evolution of cognitive processing over time. The first observation was the continued utilization of networks during the Stroop task (Run 1 = Run 2 with no changes). Networks 1, 2, 11, 12, and 13 did not show altered inter-network connectivity. These components represented the salience network (1), language network (2), visual network (11), and DMN (12, 13), which demonstrated similar activity in both runs throughout the Stroop task and were consistent with networks previously implicated in cognitive interference during this task (Peterson et al., 2002; Kalanthroff and Henik, 2014; Perrotta et al., 2021; Banich, 2019; Bush et al., 1998; Banich et al., 2000; Milham et al., 2003; Carter et al., 2000; Pardo et al., 1990; Gruber et al., 2002; Bianco et al., 2021; Ghahremani et al., 2015; Aron et al., 2014; Bari and Robbins, 2013; Song and Hakoda, 2015).

Dominant early connectivity (Run 1 > Run 2) may have been required for the appraisal of task difficulty and setting up cognitive strategies for task completion. The right angular gyrus, which integrates visual and language functions (ICA 6 and 14), and the frontal pole of the ventral attention network (ICA 6 and 8) were dominant in Run 1 and then were replaced by precentral and postcentral nodes in ICA 5, 14, and 15 in Run 2. The right frontal pole and right lateral prefrontal cortex, regions involved in response inhibition (Aron et al., 2004) and the suppression of distracting sensory information, were more activated during Run 2 for ICA 9. The frontal pole is utilized to solve problems of cognitive interference (Zysset et al., 2001). The cerebellum–fusiform gyrus connection was elevated during Run 2 for ICA 8. These changes suggest the utilization of system integration and surveillance functions of the angular gyrus and VAN early in the task (Run 1) that were replaced by object recognition by fusiform and motor preparation by SMA and motor cortex nodes in Run 2. Delayed engagement of the fusiform gyrus in ICA 8 was consistent with word recognition preceding a loop involving frontal amplification of dorsolateral cerebellar inhibition, which suppressed perceptual conflicts during the Stroop task (Okayasu et al., 2023). Inter-network changes in Run 2 may indicate that the task became easier, as suggested by increased connectivity between the precuneus of the DMN and the salience network in ICA 10. ICA 3’s (language) connectivity with the motor cortex in Run 1 was diminished in Run 2. The loss of these correlated nodes in Run 2 may reflect a “pruning” of unnecessary high-attentional and cognitive connections once task mastery was achieved.

Late-developing correlations in Run 2 may indicate more efficiently reorganized networks for task “learning,” “adaptation,” and increased cognitive efficiency. Alternatively, they may be recruited because of fatigue in other cognitive circuits. However, this explanation is less likely in these healthy control participants because there was no decline in Stroop response times or accuracy in Run 2 compared to Run 1.

Overall, the changes in inter-network connectivity suggest a shift from high awareness and analytical observation by the ACC, SMG, and inferior frontal pole nodes of the salience and ventral attention networks to more automatic recognition and motor performance by the fusiform gyrus, SMA, and paracentral nodes of the sensorimotor network. We propose that the shift from initial high-level cognitive screening to more automatic object recognition and motor control may be impaired in cognitive dysfunctions such as ME/CFS (Shan et al., 2018a; Shan et al., 2018b), long COVID, (Inderyas et al., 2026; Barnden et al., 2023; Barnden et al., 2026), Gulf War Illness (GWI; Lindheimer et al., 2021; Guerra et al., 2023; Cisler et al., 2011), and other illnesses. The 15-min protocol may also reveal effects of fatigue during prolonged testing.

The investigation was limited to healthy controls; therefore, we do not comment on comparisons with other disorders. The Stroop protocol differed from the original, classic design (Stroop, 1935) and did not investigate reverse Stroop effects (Song and Hakoda, 2015). Nevertheless, the outcomes of Run 1 (first 7.5 min) were consistent with consensus findings and neural network activation patterns observed in visual, emotional, and other forms of interference testing (Deng et al., 2018; Huang et al., 2020; Freitas et al., 2023). Data were not separated by congruent, incongruent, and neutral trials (Baraniuk et al., 2024), nor were they stratified by gender (Verhaeghen and De Meersman, 1998; Sjoberg et al., 2023).

## Data Availability

The raw data supporting the conclusions of this article will be made available by the authors, without undue reservation.
